# I Feel Less Blue When I Read With You: The Effect of Reading Aloud With a Child on Adult Readers’ Affect

**DOI:** 10.3389/fpsyg.2021.706729

**Published:** 2021-10-12

**Authors:** Sara Rabinowitz, Christina Pavlov, Brianna Mireku, Katrina Ying, Jiaqi Zhang, Kirsten Read

**Affiliations:** Department of Psychology, Santa Clara University, Santa Clara, CA, United States

**Keywords:** shared reading, reading aloud, emotions, mood, adult-child book sharing, adult-child interaction

## Abstract

This study examined the potential benefits of shared reading with a child on adult readers’ mood. In two experiments, young adults were randomly assigned to either read storybooks with a child or to read the same books aloud alone. In both experiments, readers experienced more positive emotions than those who read the story aloud alone. In Experiment 1, the level of interactivity between the reader and child also positively correlated with readers’ experience of positive emotions. In Experiment 2, participants who read with a child aligned their own book preferences with those of the child. Overall, participants preferred the longer and more complex storybook as it gave more opportunities for the reader and child to interact. These findings support the hypothesis that simple read-aloud experiences are not only positive for children, but have the potential to also positively impact the mood of adult readers who share books with a child.

## Introduction

Shared book reading with young children is a common activity in most care and early education settings. At its best, it can present a rich opportunity for interaction between an adult and child and, in turn, support engagement and learning in young children.

### Shared Reading and the Benefits for Children

A myriad of research describes the benefits for children of regular high-quality shared reading even with caregivers outside the family (e.g., teachers, librarians, and volunteers). Shared reading promotes children’s early language growth (Reese and Cox, 1999; Fitzgerald et al., 2018) and preliteracy skills (Foorman et al., 2002; Brown, 2014), assists children’s socio-emotional development and communication skills (Bergin, 2001; Aram and Shapira, 2012), and helps establish a bond between caregivers and children (Blumberg and Griffin, 2013). Dialogic reading intervention where adult readers are encouraged to actively prompt children with story-related questions as they read-aloud have been repeatedly shown to improve the language comprehension, vocabulary, and print awareness among children who are read to by adult volunteers (e.g., Fitzgerald et al., 2018). Children’s active participation, as well as the adults’ facilitation of such verbal interactions, makes shared reading especially helpful (e.g., Whitehurst et al., 1988; Zevenbergen and Whitehurst, 2003).

Shared reading is also beneficial for children’s emotional and social development. Aram and Shapira (2012) found that the more mothers reported reading with their children, the more empathetic their children were found to be. Placing a greater emphasis on the socio-emotional aspect of the story leads children to develop both their vocabulary and understanding of emotions; thus, shared reading offers children valuable socio-emotional lessons (Thompson et al., 2004; Tsai et al., 2007).

The relationship between storybook reading and the emotional benefits of shared reading for the child (and possibly also the adult reader) can be attributed to the triangular nature of shared reading (e.g., Read, 2014), such that children can benefit from both the book and the adult reader, while also being affected by how the reader interacts with the book. Given that, it is also important to consider how different book types provoke different levels of interactivity between the child and the reader (Haden et al., 1996; Nyhout and O’Neill, 2013; Muhinyi et al., 2020). Research involving shared reading interventions has found that distinct book types can be more engaging and provoke more dialogue (Muhinyi et al., 2020). Various elements of a story (e.g., the inclusion of a false belief narrative, the opportunity to make predictions, and the genre of the book) are deemed as more complex or abstract and facilitate more conversation beyond the text (Price et al., 2009; Muhinyi et al., 2020). Thus, while previous research shows that different book types inspire different levels of engagement, less is known about whether different styles of books might impact children or readers’ emotions.

### Shared Reading and the Benefits for *Both* Children and the Readers

While shared reading supports children’s early language development and socio-emotional development, research has also revealed that shared reading can help establish a bond between the child and the reader (Bus and van Ijzendoorn, 1997; Lariviere and Rennick, 2011; Blumberg and Griffin, 2013). Seden (2008) found that reading children’s books with a child supports positive parent–child relationships for low-income and stressed parents by enhancing parents’ abilities to tend to their child’s needs and empathize with their child more deeply. Blumberg and Griffin (2013) introduced a successful reading program, *Family Connections*, in adult correctional facilities, where incarcerated parents recorded their readings of storybooks and sent them to their children. Parents reported that the reading program provided them with an opportunity to re-establish their parental roles and bond with their children. Similarly, in a program for incarcerated mothers in Canada, reading aloud for their children encouraged communication, strengthened mother–child relationships, and improved the mothers’ sense of self-worth (Brown et al., 2019). Thus, shared reading can provide opportunities not just for the education of young children, but also to strengthen the emotional bond with their caregivers as well.

### Emotional Experiences in Adult Readers

The emotional connection that shared reading can build between children and adults can be affected by the emotions one experiences in adulthood. Once an individual reaches adulthood, there may be a shift in how they experience their emotions (Holodynski and Seeger, 2019). Experiences promote feelings that fluctuate between positive and negative, and these are considered in totality when taking our own “well-being” into account. For example, an individual with more consistent positive than negative experiences has a higher well-being. Because emotions depend heavily on our experiences, they are susceptible to change with each new experience. Reading aloud with a young child may be one experience that elicits positive emotional changes. Research shows that reading literature (even alone) can be a positive experience for the reader (Poerio and Totterdell, 2020) and that individuals who frequently read for pleasure are less stressed, depressed, and lonely (Rane-Szostak and Herth, 1995). Poerio and Totterdell (2020) found that listening to audiobooks had the potential to increase an individual’s sense of well-being for up to two weeks. Researchers concluded that books alone were not the sole factor that affected readers’ emotions, but the interactive experience an adult reader had with a story and how engaged they were determined their sense of well-being. Thus, reading and engaging with stories can, on its own, be a positive emotional experience, but what about reading stories and engaging *with* a younger partner?

### Shared Reading and Its Potential Benefits for Adults

Surprisingly, little research has documented the overall effects on mood for adults simply interacting with young children. While anecdotally many people find spending time with children to be a positive experience, we aim to measure the specific effects of reading with children on the adult reader, knowing already how beneficial the experience is for the young listeners. Research on the impact of shared reading experiences on the reader has been limited thus far to studies pointing to the relationship between shared reading and parent–child bonds (Bus and van Ijzendoorn, 1997; Lariviere and Rennick, 2011). Bus and van Ijzendoorn (1997) found that infants and their mothers who engaged in shared reading were more securely bonded, but because this finding is correlational, it is unclear whether shared reading facilitates a closer bond between the dyad or whether those who already have a close bond are more likely to engage in shared reading. Canfield et al. (2020) examined how shared reading can reduce stress for the adult reader. However, findings present the same problem of directionality – while they suggest that reading and reduced stress levels are correlated, we cannot assume that the reading experience is the mechanism causing a reduction in stress.

The goal of the present research was to explore whether shared reading elicits an emotional reaction, perhaps even positively, from adults who participate in these shared activities. Moreover, given that parents have an ongoing close relationship with their children, we can work toward answering this question of directionality by replacing the parent with a volunteer who does not have an established relationship with the child. Could reading aloud with a child have an immediate emotional effect on a volunteer adult reader?

### The Present Research

The current study investigates the benefits of shared reading for the adult reader and the child with two complementary experimental approaches. It is often assumed that reading to a child is a pleasant experience, but that is hardly generalizable given the variety of contexts, levels of familiarity, and even book types that can occur in any given shared reading event. We do not yet know what specific positive effects a shared reading experience may or may not provide for an adult reader and in particular for adult readers who have not established rapport with the child. In the first experiment presented here, the aim was to focus on examining possible mood effects for young adult readers in a controlled setting (e.g., reading the same set of books with the same child in the same room) in order to isolate just the possible correlation of reading with a child and mood. In the second experiment, we extended the research questions into a more natural setting, even as that introduced more variability (e.g., from the children being read to and the more distracting preschool setting) in order to strengthen the ecological validity of the findings. With these two experiments together, this study examines the possible effects of shared reading experiences on the mood and quality of interaction experienced by a young adult volunteer reader, as well as other factors that may play a role in mediating those effects.

## Experiment 1

### Method

#### Ethics

The experimental protocol described below was approved by the Santa Clara University Institutional Review Board for Social Behavioral Educational Research (protocol ID: 17–05-970). Consent was obtained from each participant before any information was collected.

#### Participants

The participants consisted of 35 undergraduate students (12 male and 22 female, 1 unknown) ranging in age from 18 to 21years. Participants were recruited using an Introductory Psychology subject pool, which allows students enrolled in introductory psychology courses to participate in research studies in exchange for required course credit. Participants were diverse in race and ethnicity, and while many participants were fluent in other languages, given their status as college undergraduates, all were proficient enough in verbal and literate English to comfortably participate. All of the participants were randomly assigned to one of three conditions: reading with child (RWC), puzzling with child (PWC), or reading aloud alone (RAA). All participants self-reported at least some level of comfort with both reading aloud and interacting with children. Undergraduates in all three conditions participated in the study during a late-afternoon thirty-minute window.

#### Materials

All participants were administered an initial background survey prior to the session and a momentary mood questionnaire Affect Valuation Index (AVI; Tsai et al., 2006) after the session (both described below). In the RAA and RWC conditions, participants were instructed to read four books used both in piloting and in previous research as well as widely available and familiar to preschool-aged children generally intended to represent typical storybook styles: a simple lighthearted children’s book about animal sounds (Sandra Boynton’s *Moo Baa La La La*), a rhyming/guess-what-comes next book about animals (Laura Leuck’s *For Just 1Day*) that has been demonstrated to provoke reader-child interaction in previous research (Read et al., 2014), a playful nonsense rhyme book *One Fish, Two Fish, Red Fish, Blue Fish* by Dr. Seuss, and a wordless picture book also used in previous research (Luo and Tamis-LeMonda, 2017) to provoke reader-child dialogue (Mercer Mayer’s *Frog Where Are You?*). In the puzzling with child condition (PWC), two 24-piece animal puzzles, which typically took the child confederate about 15min to complete, were provided to the dyad to put together in whichever order they preferred. Sessions were recorded using a digital camera placed on a table approximately 3ft. from both the couch and the child-sized table where the reading and puzzling activities occurred.

#### Procedure

Participants were instructed to meet the researcher at the laboratory space located on campus designed for testing children with child-appropriate furniture, toys, and a couch. Upon arrival, the researcher informed participants that the purpose of the study was to assess different kinds of books and activities that can best hold a child’s attention. After giving consent to participate and completing a background questionnaire, participants in the control (RAA) condition were instructed to read the storybooks as if they were reading to a child. Participants in the RWC and PWC conditions were introduced to a 5-year-old boy who served as the child confederate. In the RWC and PWC conditions, while the researcher went in an adjacent room to retrieve the materials, the participant and the confederate had a 1- to 2-min opportunity to “warm up” to each other, typically with some friendly conversation before beginning the shared reading or puzzling activities. In all three conditions, the researcher left the participant (and child confederate) to read/puzzle alone, while waiting and listening in an adjacent control room, in order to decrease observational pressure and encourage more natural interaction with the child. After reading or completing the puzzle with the child in the RWC and PWC conditions, the researcher came into the testing room and thanked the child for participating who then said goodbye and left with his mother. Once the child exited the room, the participants were administered the momentary mood questionnaire. Before concluding the session, participants were debriefed and asked whether they had any further questions or comments.

#### Measures

Measures for analysis were derived from both the pre- and posttest questionnaires and also from transcriptions that were made from the RWC and PWC condition sessions. The verbal interactions from the video recordings in the RWC and PWC condition sessions were transcribed from the moment the researcher left the room and coded for the number of extra-textual words used by the reader overall, and the number of conversational turns (i.e., back-and-forth verbalizations in response to the same content or to each other) between the child and adult participant (e.g., Gilkerson et al., 2017). These measures were intended to numerically capture the “interactiveness” of the sessions.

##### Pretest Questionnaire

Participants were asked to complete a background questionnaire that assessed participant’s frequency and comfort with reading to children, interacting with children, and reading aloud. They were asked Likert scale questions including “On a scale of 1 (*never*) to 5 (*frequently*), in the past year how often have you read to a child?.” This latter question was used as a measure of participants’ prior experience with shared reading.

##### Posttest Questionnaire

Directly after participants finished reading the storybooks or completing the puzzles, participants in all three conditions were administered a mood questionnaire, a subtest of the (AVI; Tsai et al., 2006). Participants were asked to rate their mood pertaining to how they were actually feeling at the time. The questions that the participants answered were based on 37 different emotion words. They were instructed to report their mood by rating each of these words on a scale from 1 (*not at all*) to 5 (*extremely*). These emotion words fall into 1 of 4 categories, coded as either positive (e.g., *content, happy, satisfied)*, negative (e.g., sad, unhappy, lonely), high arousal (e.g., astonished, surprised), or low arousal (e.g., *idle, passive, inactive*). Using participants’ numerical ratings, a score for each mood category was determined for each participant by adding the ratings for each of the six words per category. This yielded four mood scores (positive, negative, high arousal, and low arousal) that could each range from 6 to 30, with higher values indicating a stronger self-report of that mood.

### Results

#### Condition Effects on Participants’ Mood Scores

Descriptive statistics of participants’ mood scores in each condition are presented in Table 1. As can be seen from Table 1, participants who read-aloud with a child had the highest average positive mood and high arousal scores and the lowest negative mood scores. In order to test the statistical significance of effects of each condition (i.e., whether they were reading or puzzling with a child vs. RAA) on readers’ mood scores, a series of one-way ANOVAs were conducted with condition (RAA, RWC, or PWC) as a between-subjects independent variable and each type of mood score (positive, negative, high arousal, and low arousal) serving as the dependent variable in successive analyses. We found no significant effect of condition on participants’ low arousal mood scores, *F*(2, 31)=0.10, *p=0*.910, *eta^2^*=0.01, nor on their negative mood scores, *F*(2, 31)=2.49, *p=0*.100, *eta^2^*=0.14. However, there was a marginal effect of condition on participants’ high arousal mood scores, *F*(2, 31)=2.90, *p=0*.070, *eta^2^*=0.16, and a significant effect of condition on participants’ positive mood scores, *F*(2, 31)=5.28, *p=0*.011, *eta^2^*=0.25. *Post hoc* tests revealed this effect was driven by significantly higher positive mood scores in the RWC condition compared to the control (RAA) condition, *p*=0.014, and marginally higher positive mood scores in the PWC condition compared to control, *p*=0.062, while there was no significant difference in positive mood scores in the RWC or PWC conditions, *p*=0.817. Thus, after reading aloud with a child, participants reported higher positive feelings compared to participants who had read-aloud alone, but not necessarily compared to those doing another goal-directed interactive activity, such as a puzzle with a child.

**Table 1:** Postsession Mood Scores for Each Condition in Experiment 1.

*Mood Score*				
Condition	Positive *M* (*SD*)	Negative *M* (*SD*)	High Arousal *M* (*SD*)	Low Arousal *M* (*SD*)
Reading Aloud Alone	25.9 (5.3)	14.1 (3.9)	16.9 (4.4)	21.5 (4.7)
Puzzling with Child	31.6 (6.4)	13.5 (4.2)	20.2 (4.3)	20.5 (6.4)
Reading with Child	33.2 (6.1)	10.8 (2.7)	21.7 (6.5)	21.0 (5.9)

#### Exploratory Analyses: Readers’ Prior Experience, Interactivity, and Mood Scores

In order to better understand how participants’ previous experiences with children might have affected their mood after reading aloud or puzzling with a child vs. simply reading aloud, we split participants’ responses by condition and conducted simple bivariate correlations between participants’ reported frequency and comfort reading with young children and each of their postsession mood scores. We found that for participants in the RWC and RAA conditions, there were no significant correlations between participants’ prior experience with children and any of their mood scores after the session (all *r*’s<0.22, all *p*’s>0.450). However, for participants in the PWC condition, prior experience with children did positively correlate with participants’ subsequent positive mood scores, *r*=0.69, *p*=0.029, though not with any other mood score (all other *p*’s>0.200). Thus, anyone reading aloud with a child can experience a positive mood, but in order for a puzzling interaction to result in positive mood scores, it helps to have prior experience interacting with young children.

In a second exploratory analysis, we examined whether the interactivity between shared activities with a child affected participants’ mood scores. We also conducted bivariate correlations analyses between the amount of extra-textual talk, the amount of conversational turns participants used while reading, and their four postsession mood scores. We found that for participants in the puzzling condition, there were no significant correlations between how interactive they were with the child (in either total amount of talk or conversational turns) with any of their postsession mood scores (all *r*’s<0.35, all *p*’s>0.30). However, for participants who read-aloud with the child, there was a positive and marginally significant relationship between amount of extra-textual talk and participants’ positive mood scores (*r*=0.58, *p* =. 076). This hints that higher levels of interactivity between the adult reader and child may be connected to more positive feelings.

Qualitatively, participants in the RWC condition seemed to enjoy the storybooks that they read with the child. While participants were not explicitly asked about their preference for one particular book, one participant did report preferring *For Just 1Day*, for example, “That one had cooler rhymes.” This motivated us to ask participants to elaborate on their own book preference and the book they believed the child found most enjoyable in Experiment 2.

## Discussion of Experiment 1

The main finding of Experiment 1 was that college students who were given an opportunity to read-aloud for 10–15min to an unfamiliar 5-year-old experienced more positive emotions than those who read the same children’s stories aloud alone. Further, the correlation between amount of verbal interaction that took place within a reading session and readers’ positive mood scores suggests there may be a mediation effect, such that readers who engage in more interactive dialogue with a child during a read-aloud might experience greater positive mood boosts. Experiment 1 provides insight into how reading aloud with a child relates to the readers’ mood and highlights the triangular nature of shared reading where the child, the adult reader, and the book, all play an important role in a dynamic reading experience. However, there were limitations to this study that we attempted to address in Experiment 2.

In Experiment 1, the same 5-year-old child acted as a confederate for every participating young adult. This provided consistency across participants, but may have caused the child to become increasingly comfortable and familiar with the stories and puzzles, which could have, in turn impacted the mood of participants – reading a book aloud that is new to both the reader and the child may produce a different experience than reading a book that the child has already heard several times. Furthermore, the use of a confederate may have complicated our interpretation of the effects of interactivity since the child may have driven the interactivity more than the reader. Therefore, in Experiment 2, we made three primary changes to address these limitations: (1) We used two instead of three conditions, narrowing in on how reading with or without a child affects one’s mood; (2) participants in the experimental condition read at a child development center on campus where it was part of a normal routine to have student volunteers participate in read-alouds with the preschool-aged children; and (3) rather than having participants read with a single child confederate, participants were randomly assigned to one of several available preschoolers (assenting to be read to) at the child development center. These changes allowed us to test the relationship between reading aloud and subsequent mood of adult readers in a more ecologically valid situation. These changes were intended to enhance the external validity of the study by generalizing the findings to a more natural volunteer setting.

## Experiment 2

### Method

#### Ethics

The experimental protocol described below was approved by the Santa Clara University Institutional Review Board for Social Behavioral Educational Research (protocol ID: 19-08-1,310). Consent was obtained from each participant before any information was collected.

#### Participants

The participants consisted of 29 undergraduate students (4 male, 25 female) ranging in age from 18 to 21years. Participants were recruited as in Experiment 1 using an Introductory Psychology subject pool. Participants were diverse in race and ethnicity, proficient in verbal and literate English, and self-reportedly comfortable interacting with children and reading aloud. Given university and preschool closures in response to public health orders due to COVID-19, the sampling for this study was less than the total number expected. Thus, the data presented are based on only the 29 participants that were able to complete testing before closures took place. All of the participants were assigned to one of two conditions: RWC or RAA. Those who signed up to participate in the study on a Monday or Wednesday were assigned to the RAA condition and those who signed up to participate in the study on a Tuesday or Thursday were assigned to the RWC condition. Undergraduates in both conditions participated in the study during a late-afternoon thirty-minute window.

#### Materials

In order to keep the reading sessions shorter and more natural, only two of the books used in Experiment 1 were used in Experiment 2: *Moo Baa La La La* by Sandra Boynton and *For Just 1Day* by Laura Leuck. A posttest questionnaire (described below) similar to that used in Experiment 1 was administered to participants after their session *via* an electronic link to an online survey form.

#### Procedure

Upon arrival, the researcher informed participants in both conditions that the purpose of the study was to examine different kinds of stories and activities that can best hold a child’s attention. To ensure that participants in the experimental (RWC) condition were unaware that they would be going to a child development center upon signing up for the study, participants were first met at a central location on campus within a short walk to the center. Before beginning their read-aloud, participants in the experimental (RWC) condition were reminded of the general rules of the preschool environment (e.g., in an effort to respect a child’s choice to listen to the story, if they are no longer interested, they are allowed to go and play). At the child development center, the participant was given two children’s books and was assigned to read to a preschool-aged child by the class teacher who knew which children were eager for a story and had or had not participated previously. The participant read the two children’s books to the child in the designated outdoor garden space during their scheduled free play time.

Participants in the control (RAA) condition were asked to read-aloud the same two books in the same laboratory space where Experiment 1 took place. Different from the experimental (RWC) condition, participants in the control condition were without the presence of a child listener to interact with. After providing written consent, participants were instructed to read the two children’s books aloud as if they were reading to a child who may later listen to the recording as they follow along with a print version of the story. In an effort to further motivate participants to actively engage with reading the stories as they would if a child was present, they were told (falsely) that the process was being recorded for a child to hear later, with the use of a digital recorder serving as a prop. At the end of the reading, as per our debriefing protocol, participants were told that they had not actually been recorded, but their read-alouds were still helpful for our research. No complaints were made about this minor deception.

#### Measures

Measures for analysis were derived from the posttest questionnaire.

##### Postreading Survey

After participants read the two children’s storybooks, they were asked to complete a postreading survey electronically *via* an anonymous survey link. Participants in the RWC condition completed the survey in a quiet staff room at the child development center, and participants in the RAA condition did so at a table in the laboratory space. This postreading survey included the same momentary mood questionnaire as used in Experiment 1, to derive measures of each participant’s postreading levels of both positive/negative emotions and high/low arousal.

Additionally, measures of book preference and interactivity were derived from the second part of the postreading survey. All of the participants in this study were asked to rate how engaging they believed their reading was on a scale of 1 (*not at all*) to 6 (*very*) and to indicate which book they preferred to read and which book they would consider to be the most effective in keeping a young child engaged. Participants in the experimental (RWC) condition were asked an additional set of questions regarding their level of interactivity with the child during their shared reading time. Participants were asked questions including “On a scale of 1 (*never*) to 6 (*frequently)*, how often did you have side conversations during the shared reading?” and were asked to share a positive or memorable interaction they experienced which made them feel good. After completing the survey, participants in both conditions were debriefed on the study and asked whether they had any further questions or comments about their session before leaving.

### Results

#### Condition Effects on Participants’ Mood Scores

Descriptive statistics of participants’ mood scores in each condition are presented in Table 2. In order to test whether there were effects on readers’ mood scores, we conducted a series of four independent samples t tests to analyze the mean reported positive, negative, high arousal, and low arousal mood scores across participants in each condition. We found no significant effect of condition on participants’ low arousal mood scores, *t*(27)=−0.88, *p=0*.385, *d*=0.33, nor on their negative mood scores, *t*(27)=−0.99, *p=0*.311, *d*=0.37. There was, however, a moderate effect approaching significance in their positive mood scores, *t*(27)=1.56, *p=0*.131, *d*=0.58, and a significant effect of condition on participants’ high arousal mood scores, *t*(27)=2.13, *p=0*.042, *d*=0.80, such that participants who read-aloud with a child were feeling somewhat more positive and more excited than those who simply read the same books aloud for the purpose of a recording.

**Table 2:** Postsession Mood Scores for Each Condition in Experiment 2.

*Mood Score*				
Condition	Positive *M* (*SD*)	Negative *M* (*SD*)	High Arousal *M* (*SD*)	Low Arousal *M* (*SD*)
Reading Aloud Alone	27.1 (6.2)	14.1 (3.5)	17.9 (4.1)	21.4 (5.1)
Reading with Child	31.1 (7.4)	12.6 (4.4)	21.5 (4.8)	19.9 (3.9)

#### Correlations Among Background, Reading Interactivity, and Participants’ Mood Scores

In order to explore whether participants’ previous experiences with children might have affected their mood after reading aloud with a child vs. simply reading aloud, we split participants’ responses by condition and then conducted simple bivariate correlations between participants’ reported comfort with young children and each of their postsession mood scores. We found that for participants in both the RWC and RAA conditions, there were no significant correlations between participants’ prior experience with children and any of their postsession mood scores (all *r*’s<0.32, all *p*’s>0.300). Additionally, to test whether there were any relationships between how engaging participants believed their own readings to be and their subsequent mood scores, we also split responses by condition and then conducted simple bivariate correlations between how engaging participants self-reported their read-alouds to be and each mood score, finding again that there were no significant correlations in either condition (all *r*’s<0.35, all *p*’s>0.240). Lastly, in the RWC condition, there was also no significant correlation between participants’ ratings of how interactive their reading sessions had been and any of their four subsequent mood scores (all *r*’s<0.35, all *p*’s>0.230).

Finally, a further exploratory analysis was conducted to better understand whether there were differences between the participants’ book preferences and their beliefs about which books the children preferred. We found that overall participants reported a stronger preference for the longer storybook, *For Just 1Day*, with 21 out of 29 participants (72%) indicating that was the book they most enjoyed reading. Additionally, most participants (22 out of 29, or 76%) believed that the child they read with also preferred *For Just 1Day* over the other book. Most notably, 24 out of 29 participants (83%) believed that the book they preferred was the same one that the child preferred regardless of which book that was, illustrating a strong alignment between the readers and the children.

## Discussion of Experiment 2

The main finding of Experiment 2 was that college students who were given an opportunity to read-aloud to a newly introduced preschooler experienced more excitement and somewhat more positive feelings than those who read the same children’s stories aloud alone. These findings echo those of Experiment 1.

Unfortunately, due to challenges presented by COVID-19, the sample size was limited to 29. Cancellation of all on-campus operations for undergraduate students and the closure of the on-campus child development center began in March 2020 and remained throughout the year. We made the decision to end enrollment in the study rather than wait out the closures because it was impossible to know how long the delay would be and also to what extent the pandemic would have impacted the emotional climate for volunteers reading with children on campus after such a delay. COVID-19 would certainly have caused history effects in the data that we were not prepared to overcome.

The changes that were made to Experiment 2 in order to address the limitations of Experiment 1, however, did broaden the generalizability of these findings even without a large sample. Firstly, we narrowed down the study to two conditions – the RWC and RAA conditions – for the purpose of increasing power and direct comparison, and we streamlined the reading session to just two books. Also, in Experiment 2, the RWC condition was conducted at the child development center instead of at the laboratory space and with a rotation of child listeners rather than a consistent confederate. This provided a more natural setting for shared reading and interacting with children. With this more focused comparison in a more natural setting, we found that similarly to Experiment 1, participants reported a higher mood boost when reading with a child compared to participants reading aloud alone. In general, participants in Experiment 2 preferred the longer, *For Just 1Day* storybook as well, perhaps because it allowed for more opportunities for the participants to interact and converse with the children. Thus, even with a less-than-ideal sample size and the greater variability introduced in the more natural setting with a more varied group of children, we still found evidence of a link between reading aloud with children and the positive experience for the readers.

## General Discussion

### Implications for a Positive Mood Boost

Taken together, both experiments reveal how reading with a child present can relate to the mood of the adult reader, whether encouraging the reader’s feelings of excitement or positivity. In Experiment 1, college students who were given an opportunity to read-aloud for 10–15min to an unfamiliar 5-year-old experienced more positive emotions than those who read the same children’s stories aloud alone. In Experiment 2, college students who were given an opportunity to read-aloud to a newly introduced preschooler experienced more excitement and somewhat more positive feelings than those who read the same children’s stories aloud alone. Aside from the PWC condition in Experiment 1, in neither experiment did participant’s prior experience with children correlate with their mood scores after the reading session. And, in both Experiments, there were signs of positive effects of interactivity and alignment between the reader and child. These findings suggest that there is indeed something measurably positive about reading aloud with a child. While there are limitations to the power of our study, we see marginal effects of reading aloud on both the participants’ positive mood and on how energized they feel after reading aloud with a child. Building on previous studies that have found correlations connecting shared reading and positive parent–child bonds (Bus and van Ijzendoorn, 1997; Lariviere and Rennick, 2011; Blumberg and Griffin, 2013), this study tests a single-session effect of reading with a child without a preexisting relationship and the subsequent positive mood boosts that may result.

### Shared Book Preferences and Reader-Child Engagement

One finding of note in this study was that the overall preference for the children’s book *For Just 1Day* suggests that specific features of the book were favorable to *Moo Baa La La La*. When asked to elaborate on their book preference, participants who favored *For Just 1Day* reported preferring the book because it was longer and prompted more interactions with the child in engaging and specific ways. By presenting the child with more opportunities to guess which animal came next, this significant feature of the book provided more room for spontaneous language play and commentary between the adult reader and child. Most participants not only preferred *For Just 1Day*, but reported feeling that the child also preferred this book. Specifically, some participants reported preferring a given book because they felt the child seemed to enjoy it more, for example, “I liked the second book because it was more interactive and the little girl seemed to enjoy it more” “and Definitely the second book because she kept smiling when I was reading and also giggled.” Some participants even directly commented on how the reading experience affected their mood, for example, “The child seemed to be very into the story and made comments about the pictures which were very cute and interacting with him made me feel good” and “I was having an awful day and honestly reading these books aloud made me drift from that bad mood even if it was just for a little while.” The strong alignment between the reader and child’s book preference points to the triangular nature of shared reading (e.g., Read, 2014). Sharing a preference for the same book is a dimension of a shared experience and suggests that there may be an emotional tie between the adult reader and child.

### Generalizability: Limitations and Future Directions

Because of the challenges of recruiting available young children in natural settings for shared reading experiences and the specific challenges presented by COVID-19, both experiments in this study were limited in sample size. The smaller sample size makes it harder to overcome internal variability, and yet variability is what makes the shared reading experiences most natural. Thus, future research building on these findings is needed to increase the power of these measures and analyses. More specifically, future research could take advantage of established, multi-site, or longer-term reading volunteer programs – enabling more young adult populations beyond university students to participate as well as a further investigation of changing mood effects over time. Involving a more diverse sample in this way would improve the generalizability of these findings. Additionally, replicating this work with a larger and more diverse sample would allow a closer look at how individual differences in the personalities or initial moods of the participants might impact the extent to which shared reading with a child promotes positive changes for readers. While a presession mood or personality questionnaire was not administered in the current studies because of an effort to avoid demand characteristics, future work could include more “baseline” measures of such factors for each participant in an effort to more precisely determine the magnitude of a potential mood boost for young adults reading with young children.

Building out this work with future studies would also allow us to look more closely at the mediating effects of interactivity on the emotional impacts of shared reading on adult readers. While in Experiment 1 the amount of extra-textual talk was linked to participants’ positive mood scores in the reading conditions, the findings of Experiment 2 revealed that participants’ ratings of how interactive their reading sessions had been did not relate to any of their postsession mood scores. Future research should investigate whether a stronger measure of interactivity would reveal larger effects of a positive mood boost and even the direction of that effect – does more interactivity cause a better mood or does being in a better mood promote more interactivity?

The present study adds to the work done by Canfield et al. (2020) by focusing on adult participants who have no preexisting relationship to the child. By including participants, such as these, the findings are generalizable to a more natural volunteer setting. This raises the question of whether the benefits to the adult reader are merely short term or are long-lasting. If reading sessions in the RWC condition were repeated, would this mood boost aggregate or is the effect on mood fleeting? It is important to consider the longer-term implications of this mood boost, perhaps with longitudinal studies that measure mood over repeated volunteer experiences across multiple weeks or months.

As previous studies have already examined the differences between how adults regulate their emotions, future areas of study should also probe differences in the potential mood boosts from shared reading between younger and older adults. Burr et al. (2020) reported that older adults experience more stability in their emotions and have an easier time balancing their desires and stressors because they remember more positive rather than negative stimuli. By contrast, young adults have been found to “hold onto” their negative experiences due to the way that they regulate their emotions. (Burr et al., 2020). Given that we included only young adults in this study, we can only hypothesize that this developmental change in emotion regulation might moderate the emotional effects of sharing a book with a young child. Future research could continue to expand this work into age groups such as middle-aged adults with or without experience with children under their own care and older adults who may possibly experience bigger positive mood boosts when reading to a child than younger adult readers.

### Additional Factors to Consider: Limitations and Future Directions

Additionally, with larger and more diverse samples, limitations in the current study’s ability to analyze other individual difference factors could be addressed. With a broader sample, future research could investigate how the gender of the reader, the gender match with the child, and even personality traits of the adult readers may affect both how interactive and how positively they feel when reading with a child. Research examining the extra-textual verbal interactions around shared reading experiences between parents and their children has found gender pairing effects, such that mothers and fathers engage differently when reading with their sons vs. their daughters (Vandermaas-Peeler et al., 2012) and that differences among mothers in traits such as empathy can impact the amount of extra-textual interaction they have with their children while shared reading (Rollo and Sulla, 2016). If these types of individual difference variables in parents impact the interactivity of read-alouds, then given the findings in current study, they may, in turn, impact the mood associated with shared reading that the reader experiences. Thus, continued work in the area could broaden our understanding of what factors have the potential to enhance or diminish the emotional effects of reading with a child.

In examining the way shared reading impacts the adult reader, Canfield et al. (2020) discussed family systems theory, which postulates that the parents, the child, and the environment all mutually influence one another. Similar to the triangular nature of shared reading (Read, 2014), the Family Systems Model theorizes that the child also brings something to the reading experience, and in this case, may be the factor that boosts the mood of the adult participant. Our research extends this theory beyond a family to the simple shared reading interactions that happen even when a young adult volunteer and a newly introduced child read together for the first time. Thus, shared reading interactions are clearly non-stagnant. Situational and emotional variables can change from one shared reading experience to another and cannot exist in isolation from each other. Altogether, this work illustrates that reading aloud is a dynamic process that can affect the reader as well as the child.

## Data Availability Statement

The raw data supporting the conclusions of this article will be made available by the authors, without undue reservation.

## Ethics Statement

The studies involving human participants were reviewed and approved by Santa Clara University Institutional Review Board for Social Behavioral Educational Research. Written informed consent to participate in this study was provided by the participants’ legal guardian/next of kin.

## Author Contributions

All authors contributed to the initial question development, design, implementation, analysis, and writing of this study. In addition, KR served as faculty advisor on student work, research lead, and final editor.

## Conflict of Interest

The authors declare that the research was conducted in the absence of any commercial or financial relationships that could be construed as a potential conflict of interest.

## Publisher’s Note

All claims expressed in this article are solely those of the authors and do not necessarily represent those of their affiliated organizations, or those of the publisher, the editors and the reviewers. Any product that may be evaluated in this article, or claim that may be made by its manufacturer, is not guaranteed or endorsed by the publisher.
